# Ferrocene-Branched Polymers via a Ugi Multicomponent Reaction for an Integrated and Reagentless Amperometric Glucose Biosensor

**DOI:** 10.3390/bios16100554

**Published:** 2026-10-02

**Authors:** Jorge Urraca, Isabel Zurita, Marija Krznaric, Tatiana Martínez, Alfredo Sánchez, Diana Vilela, Paloma Martínez-Ruiz, Irene Ojeda, Reynaldo Villalonga

**Affiliations:** Nanosensors and Nanomachines Group, Department of Analytical Chemistry, Faculty of Chemistry, Complutense University of Madrid, 28040 Madrid, Spain; jorgeurr@ucm.es (J.U.); isabel.zurita@estudiante.uam.es (I.Z.); mkrznaric1.chem@pmf.hr (M.K.); tati3520@gmail.com (T.M.); alfredos@ucm.es (A.S.); divilela@ucm.es (D.V.); palmarti@ucm.es (P.M.-R.)

**Keywords:** Ugi reaction, polyethyleneimine, ferrocene, glucose biosensor, food analysis

## Abstract

A Ugi multicomponent reaction was employed to synthesize a ferrocene (Fc)-branched polyethyleneimine derivative. This polymer was evaluated as a redox-active matrix for the fabrication of an electroanalytical disposable glucose biosensor based on a carbon PE-printed electrode functionalized with glucose oxidase as a biorecognition element. The resulting biosensor displayed a linear response up to 35 mM glucose at pH 6.5, with a limit of detection of 33 µM. The enzyme biosensor reached 95% of the steady-state current within approximately 50 s and showed a sensitivity of 0.92 µA/mM. In addition, this analytical device exhibited high selectivity, good reproducibility, and excellent storage stability, retaining strong analytical performance after 45 days at 4 °C. The proposed electrochemical biosensor was successfully applied to glucose determination in red and white wine samples.

## 1. Introduction

The determination of sugar content is a critical parameter in the analysis of wines as it is closely related to product quality, fermentation control, and sensory properties [1]. In wine, glucose and fructose are the main fermentable sugars, and their concentration provides valuable information about grape ripening, must composition, fermentation progress, and the quality of the final product [2]. In particular, glucose, as one of the major reducing sugars present in grape-derived samples, is a relevant analytical target for the wine industry. Therefore, the development of reliable methods for glucose determination is of considerable importance in enological analysis and quality control [3].

Conventionally, glucose in must and wines is determined by enzymatic assays, spectroscopic approaches, and chromatographic techniques coupled with an appropriate detection technique [4]. Enzymatic methods are widely employed because of their specificity and suitability for routine analysis, typically through UV–Vis monitoring of reaction products, although they still require reagent handling, controlled assay conditions, and laboratory-based instrumentation [2]. In parallel, Fourier-transform infrared (FTIR) and solid-state ^13^C nuclear magnetic resonance (NMR) spectroscopic techniques have attracted considerable interest for wine industry because they allow fast measurements with little or no sample preparation [5,6]. However, their application to glucose determination requires expensive equipment, and is often affected by matrix complexity, spectral overlap, and the need for robust chemometric calibration models. Chromatographic methods, especially high-performance liquid chromatography (HPLC) coupled with an appropriate detection technique, provide high sensitivity, selectivity, and reproducibility for sugar analysis [7], although they are generally time-consuming and rely on expensive instrumentation and trained personnel. Consequently, these methodologies are not always ideal for rapid, routine, or on-site glucose determination.

In recent years, the development of novel enzyme-based electrochemical sensing platforms has promoted the design of a wide variety of glucose biosensors [8,9,10]. Despite their promising analytical performance, many of these systems still involve complex fabrication procedures, sophisticated operating principles, or relatively high cost. Therefore, there remains a need for simple, rapid, and cost-effective analytical tools for glucose determination in wines and grape musts, particularly for on-site applications in wineries and wine production facilities.

In electrochemical enzymatic glucose biosensors, the use of redox mediators is a well-established strategy to facilitate electron transfer from the redox center of glucose oxidase (GOx) to the electrode surface [9,11]. This approach avoids the strong dependence of the enzymatic response on dissolved oxygen and overcomes the limitations associated with direct electron transfer. In addition, mediated systems generally allow operation at lower working potentials, which contributes to improved selectivity and sensitivity [12]. Nevertheless, the use of soluble mediators in the measuring solution presents important drawbacks, particularly mediator leaching during operation, reduced long-term stability, and the need to add the mediator as an external reagent [13]. Therefore, the immobilization or integration of the mediator within the transduction interface is highly desirable, as it enables the development of reagentless biosensors with enhanced operational stability, improved reproducibility, and greater suitability for practical analytical applications.

Accordingly, different strategies have been reported to integrate redox mediators into the transduction interface, either by direct attachment to the electrode surface or to the enzyme, or by using polymeric and nanostructured materials bearing redox-active moieties [14,15,16,17,18]. Among these approaches, hydrophilic polymers are particularly attractive for biosensing applications because they provide a favorable microenvironment for the enzyme, contribute to the structural and operational stability of the sensing layer, and enable stable covalent immobilization of the biorecognition element [17].

This manuscript describes the development of an original amperometric disposable glucose biosensor based on screen-printed carbon electrodes (SPCE) modified with a novel ferrocene-branched polyethyleneimine (PEI) derivative synthesized via a Ugi multicomponent reaction. Ferrocene (Fc) is a well-established redox mediator widely used in GOx-based electrochemical biosensors [19], whereas PEI has been extensively employed as a versatile platform for the covalent and non-covalent immobilization of biorecognition elements in electrochemical biosensing interfaces [20,21]. Within this framework, the Ugi reaction constitutes an attractive synthetic strategy owing to its simplicity, high yield, and versatility [22], particularly its ability to introduce multiple redox moieties at the polymer branching points.

The rationale behind this biosensor design lies in the incorporation of Fc units into an amino-rich polymeric transduction interface, thereby promoting efficient mediated electron transfer, eliminating the need for soluble mediators in the measuring solution, and enabling covalent enzyme immobilization. Furthermore, Nafion was incorporated into the sensing architecture as a film-forming and stabilizing component, while glutaraldehyde was used as a crosslinking agent for GOx immobilization, thereby improving the stability and robustness of the biosensing interface.

## 2. Materials and Methods

### 2.1. Materials

Glucose oxidase (GOx) from *Aspergillus niger* (EC.1.1.3.4, 250 U/mg), *t*-butyl isocyanide, Nafion^TM^ 5% (*w*/*v*), bovine serum albumin (BSA), Fc-carboxylic acid, glutaraldehyde 25% (*w*/*v*) and a glucose assay kit were acquired from Sigma-Aldrich Co. (St. Louis, MO, USA). Branched PEI, (Mw = 10,000) was purchased from Alfa Aesar (Ward Hill, MA, USA). Fc-carboxaldehyde was acquired from Acros Organics (Geel, Belgium). Screen-printed carbon electrodes (SPCEs), consisting of a carbon working electrode (4.0 mm diameter), a carbon counter electrode, and an Ag/AgCl pseudo-reference electrode, were purchased from Orion High Technologies S.L (Madrid, Spain). Chulato de Abeica red wine and Don Simón Chardonnay-Airén dry white wine were purchased from a local market. All other chemicals were analytical grade.

### 2.2. Equipment

All electrochemical studies were performed using an EmStat3 Blue potentiostat controlled with PSTrace software version 5.9 (PalmSens, Houten, The Netherlands). Fourier-transform infrared spectroscopy was conducted using an Alpha spectrometer (Bruker, Billerica, MA, USA). Elemental analysis was performed using a LECO CHNS 932 microanalyzer (LECO, St. Joseph, MI, USA), while solid-state ^13^C NMR spectra were acquired with a Varian Infinity 400 MHz spectrometer (Varian, Palo Alto, CA, USA). Thermal properties were evaluated using a SDT-Q600 thermal analysis system (TA Instruments, New Castle, DE, USA). UV–visible measurements were performed with an Ultrospec™ 8000 dual-beam spectrophotometer (Biochrom, Cambridge, UK). Surface morphology of the modified electrodes was characterized by high resolution field emission scanning electron microscopy (FE-SEM) using a JEOL JSM-7600F microscope (JEOL Ltd., Tokyo, Japan).

### 2.3. Synthesis of Ferrocene-Branched PEI (2Fc-PEI)

Branched PEI (50 mg), Fc-carboxylic acid (107 mg), and Fc-carboxaldehyde (94 mg) were added to a round-bottom flask, with PEI serving as the limiting reagent. After the addition of 30 mL of Milli-Q water (Merck Millipore, Burlington, MA, USA), the mixture was stirred until a homogeneous solution was obtained. Then, *t*-butyl isocyanide (52 µL) was carefully added in excess, and the reaction mixture was stirred for 24 h at room temperature under a fume hood. After completion of the reaction, the resulting solution was purified by dialysis against Milli-Q water using a SnakeSkinTM dialysis membrane (10,000 MWCO, Thermo Scientific, Waltham, MA, USA). Finally, the dialyzed solution was lyophilized to afford a dark-brown solid.

### 2.4. Preparation of the Enzyme Electrode

The glucose biosensor was fabricated by drop-casting a film-forming mixture onto the working electrode surface. Briefly, the cold mixture was freshly prepared by mixing BSA solution (8 mg in 100 µL of 0.1 M sodium phosphate buffer, pH 7.4), Nafion 5% solution (60 µL), 2Fc-PEI polymer solution (132 µg in 100 µL of Milli-Q water), and GOx (14.3 mg). After obtaining a homogeneous mixture, glutaraldehyde solution (40 µL, 2.5% (*w*/*v*) in water) was added as the cross-linking agent.

An aliquot of 8 µL of the resulting mixture was carefully drop-cast onto the working electrode surface. The modified electrodes were then stored overnight at 4 °C to allow the formation of a homogeneous and stable cross-linked film. After this drying/curing step, a thin, dense film remained firmly attached to the working electrode surface. The electrode was then washed several times with cold 50 mM sodium phosphate buffer, pH 6.5, dried under N_2_, and finally stored at 4 °C until use.

### 2.5. Electroanalytical Determinations

Amperometric measurements were performed by depositing 60 µL of the corresponding glucose solution onto the surface of the modified working electrode. The amperometric measurement was initiated immediately after sample addition, and a constant potential of +0.40 V was applied for 200 s while the resulting current–time response was recorded. The analytical signal was taken from the steady-state current obtained under these conditions.

### 2.6. Wine Sample Analysis

The commercial red wine sample was diluted 5-fold and the white wine sample was diluted 50-fold with 0.1 M sodium phosphate buffer (pH 6.5) prior to analysis to obtain final glucose concentrations within the working range of the colorimetric assay (0.11–0.44 mM). This dilution step also minimized the effect of the intrinsic acidic pH of the wine matrix and adjusted the sample medium to the optimized pH conditions of the biosensor. For the reference colorimetric assay, 1.0 mL of the diluted sample was mixed with 2.0 mL of the commercial glucose reagent, containing GOx, peroxidase, and *o*-dianisidine. The mixture was incubated at 37 °C for 30 min, after which the reaction was stopped by adding 2.0 mL of 6 M H_2_SO_4_. The absorbance was then measured at 540 nm by UV–Vis spectrophotometry, and the glucose concentration was calculated by interpolation from the calibration plot. Amperometric measurements were performed as previously described.

## 3. Results and Discussion

**Scheme 1:**
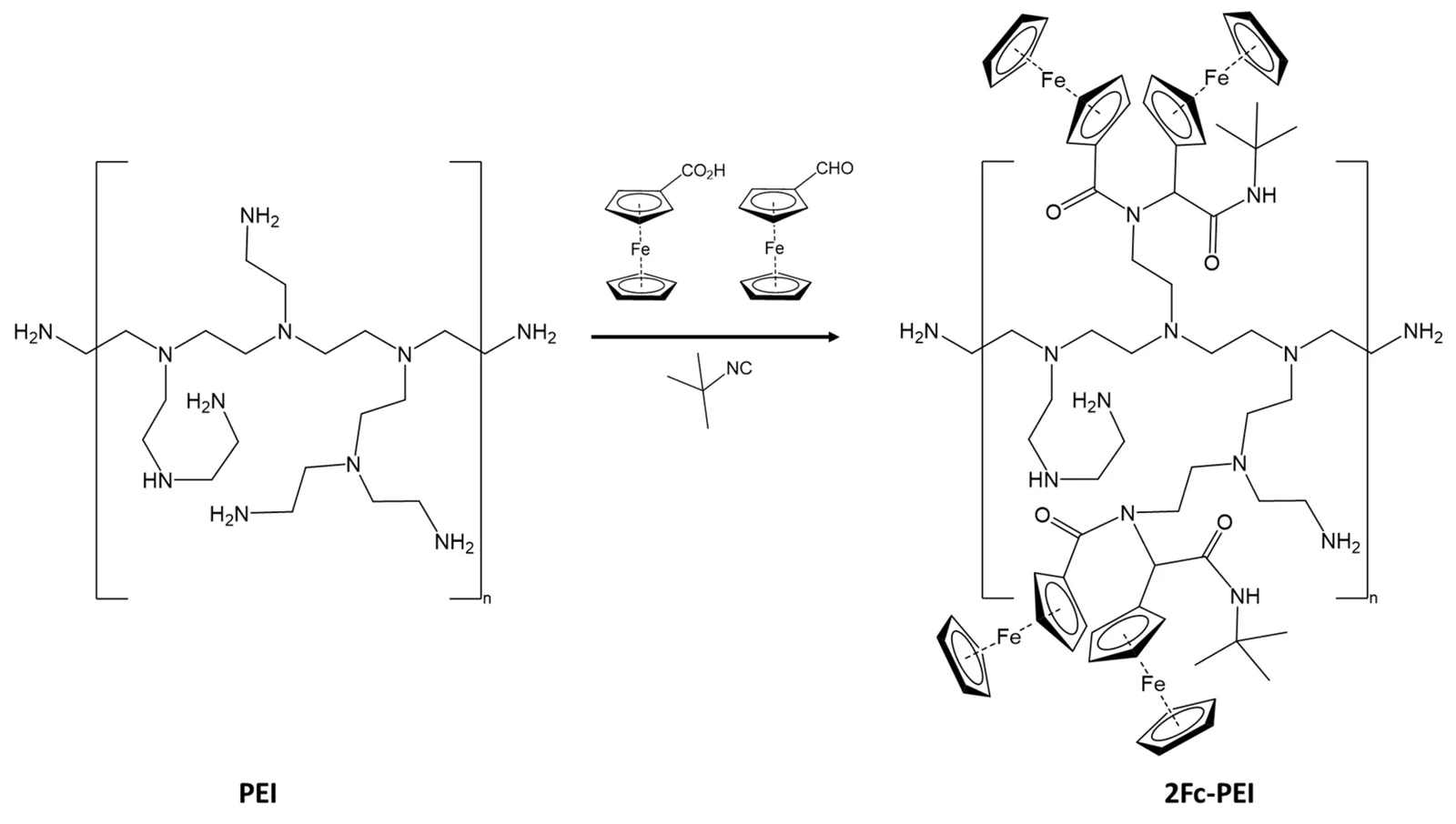
Synthesis of Fc-branched PEI (2Fc-PEI) through Ugi multicomponent reaction.

The synthetic strategy employed to prepare the Fc-branched PEI derivative (2Fc-PEI) is represented in Scheme 1. The rationale of this approach relies on the use of the Ugi multicomponent reaction for easy, controlled and one-pot incorporation of two mediator moieties at each modification point within the polymer structure. The Ugi reaction is a four-component multicomponent reaction involving an amine, a carbonyl compound, a carboxylic acid, and an isocyanide. In the classical mechanism, condensation of the amine and carbonyl component generates an imine/iminium intermediate, which undergoes addition of the isocyanide to form a nitrilium intermediate. Subsequent addition of the carboxylate and Mumm rearrangement leads to the characteristic α-acylamino amide Ugi product [22,23]. In the present work, the primary amino groups of branched PEI, Fc-carboxaldehyde, Fc-carboxylic acid, and t-butyl isocyanide act as the four reaction components, enabling the one-pot incorporation of Fc-containing Ugi adducts into the polymer structure. To achieve this, Fc-carboxaldehyde and Fc-carboxylic acid were reacted with the primary amino-enriched branched polymer in the presence of *t*-butyl isocyanide.

The incorporation of Fc moieties in the PEI macromolecular chains was confirmed by various characterization techniques. The solid-state ^13^C NMR spectrum of Fc-enriched polymer, recorded at a 12 kHz magic-angle spinning rate, showed peaks at 60–35 ppm, corresponding to the carbon atoms in the highly branched PEI (Figure S1 in Supporting Information) [24]. The prominent peak at 69.9 ppm, ascribed to the cyclopentadienyl carbons, revealed the presence of ferrocenyl groups in the modified polymer [25]. This hypothesis was also confirmed by the peaks at 79.4 ppm and 176.1 ppm, assigned to the ferrocenyl carbons linked to the polymer [26] and the carbonyl groups [27] in the Ugi ensemble, respectively.

Figure S2 in the Supporting Information shows the FT-IR spectra of the main raw materials, as well as the synthesized polymer derivative. The spectra of pristine and Fc-branched PEI derivative revealed the characteristic peaks at 772 cm^−1^ and 930 cm^−1^ corresponding to the N-H bending vibration in the main polymer chain [28]. The peaks at 1046 cm^−1^ and 1107 cm^−1^ can be ascribed to the C-N stretching vibrations, the peaks at 1295 cm^−1^ and 1350 cm^−1^ to the C-C stretching vibrations, and the peak at 1463 cm^−1^ corresponds to C-H symmetric bending vibration. In addition, the bending modes of -NH_2_ side chain in PEI were confirmed by the peaks at 1599 cm^−1^ and 1671 cm^−1^, and the C-H stretching vibrations of the methylene groups by the peaks at 2820 cm^−1^ and 2945 cm^−1^. Finally, the broad peak centered at 3320 cm^−1^ can be ascribed to the N-H stretching vibrations and adsorbed water.

On the other hand, and similarly to Fc-carboxaldehyde and Fc-carboxylic acid, the FTIR spectra of Fc-branched PEI showed the characteristics peaks at 823 cm^−1^, 1023 cm^−1^ and 1108 cm^−1^, corresponding to the C-H out of plane, C-C-H bend and cyclopentadiene breathing vibrations, respectively [29,30]. The peak at 497 cm^−1^ can be ascribed to the asymmetric ring metal stretching vibration, which is characteristic of Fc molecules. The successful synthesis of the Ugi derivative was also confirmed by the large peak at 1660 cm^−1^, corresponding to the stretching vibration of the newly formed C=O bonds in the modified polymer.

Elemental analysis revealed C, H and N contents of 51.8%, 10.6% and 29.9%, respectively, for pristine PEI. In contrast, the modified polymer showed C, H and N contents of 53.0%, 6.2% and 10.1%, respectively. The marked decrease in nitrogen content and the increase in the atomic C/N ratio from 2.0 to 6.1 are consistent with the successful incorporation of carbon-rich Fc-containing Ugi adducts onto the PEI backbone, supporting the successful functionalization of the polymer.

Thermogravimetric analysis (TGA) under N_2_ further supported the successful modification of PEI with Fc-containing Ugi adducts. As shown in Figure S3 (Supporting Information), both the pristine PEI and 2Fc-PEI exhibited an initial weight loss below ca. 120 °C, which can be attributed to the removal of physically adsorbed water and/or residual moisture. After this initial dehydration step, pristine PEI underwent a single major decomposition process, with an abrupt weight loss mainly between ca. 300 °C and 400 °C, leaving only a low residual mass at 1000 °C. In contrast, 2Fc-PEI displayed a markedly different thermal behavior, characterized by a broader, multistep degradation profile and a substantially higher residual mass. This behavior indicates that the incorporation of bulky Fc-containing Ugi adducts significantly modifies the thermal degradation pathway of the PEI matrix.

The multistep degradation profile of 2Fc-PEI suggests a more complex decomposition process after functionalization. The initial mass-loss steps following dehydration can be tentatively assigned to the release of volatile fragments, including carbon oxides and other small molecules generated from the thermal degradation of amide linkages and labile moieties within the Ugi adduct. Further degradation at higher temperatures is likely associated with the progressive decomposition of the cyclopentadienyl rings and the PEI backbone.

Notably, 2Fc-PEI retained a much higher residual mass at 1000 °C than pristine PEI. Under an inert atmosphere, this residue may consist of a combination of iron-containing species and carbonaceous residue generated during pyrolysis. These results strongly support the incorporation of Fc-containing Ugi adducts into the polymer structure.

**Scheme 2:**
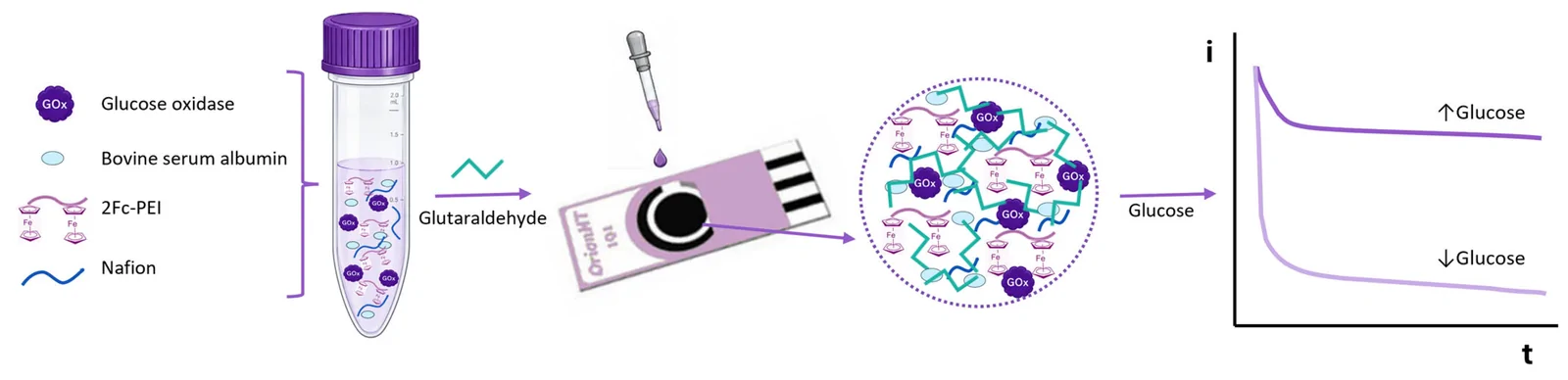
Fabrication of the GOx/2Fc-PEI-modified SPCE. The arrows in the amperometric response indicate high (↑Glucose) and low (↓Glucose) glucose concentrations.

The branched polymer served as the electrochemical transducing matrix for the fabrication of a reagentless enzymatic glucose biosensor (Scheme 2). Nafion and bovine serum albumin were included to enhance film integrity and enzyme stabilization, whereas glutaraldehyde acted as the cross-linking agent.

**Figure 1:**
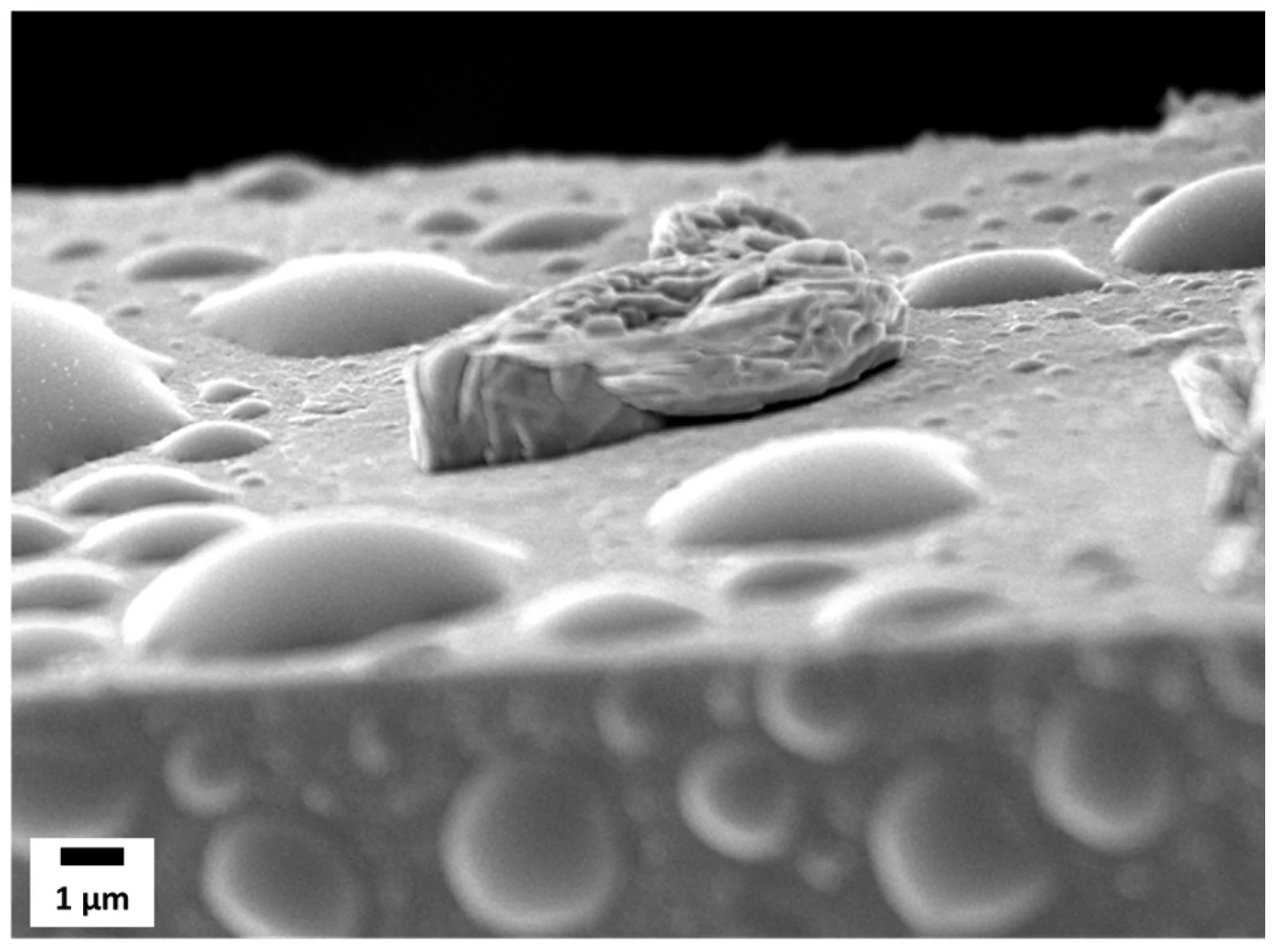
Representative FE-SEM images of the drop-casting film on the working electrode surface.

FE-SEM analysis showed a continuous micrometric coating on the working electrode surface, with globular protuberances distributed throughout the film (Figure 1). These morphological features may be associated with protein-rich domains or aggregates formed during film formation and glutaraldehyde-mediated cross-linking, in agreement with previously reported morphologies of cross-linked enzyme systems [31,32].

**Figure 2:**
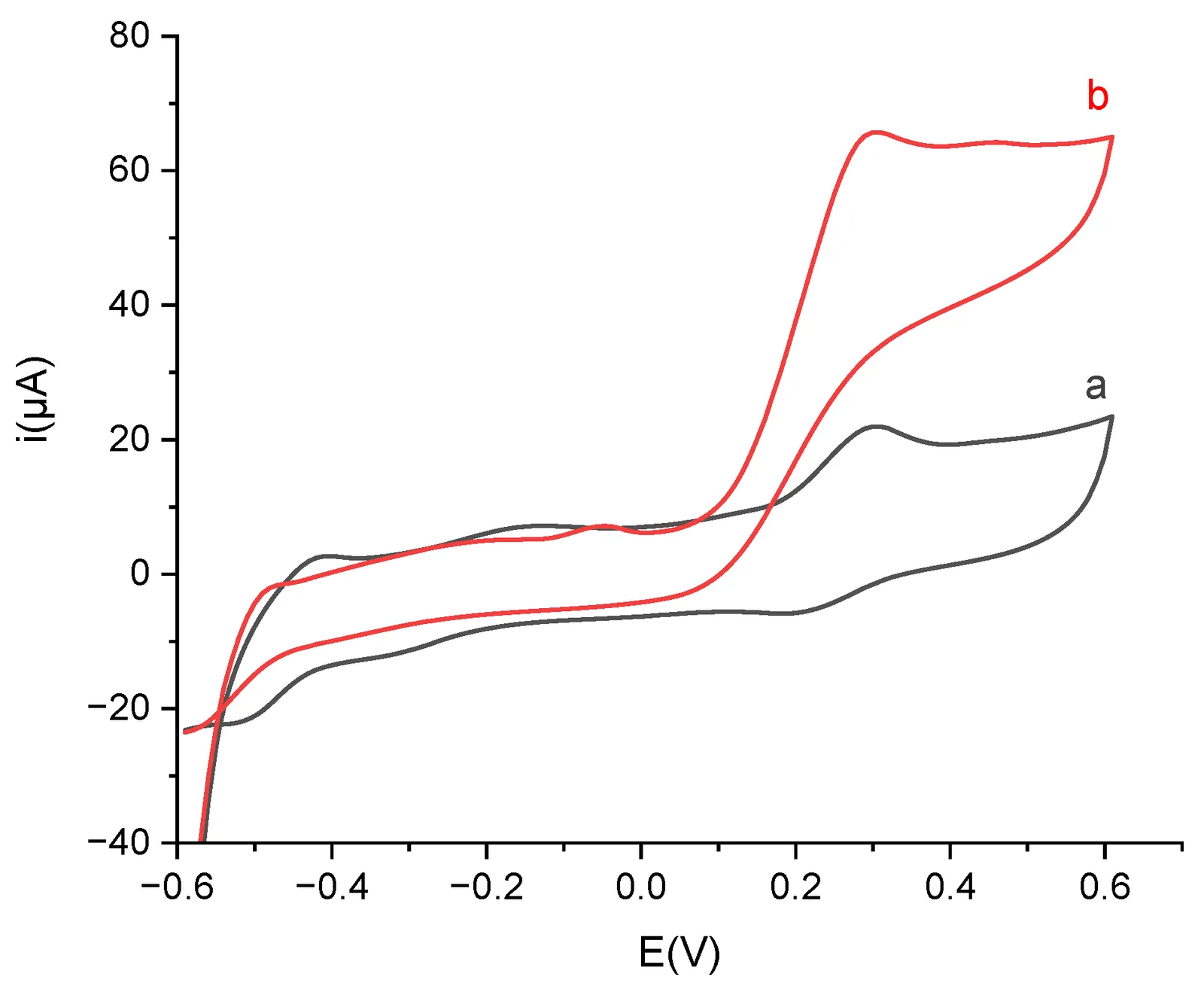
Cyclic voltammograms recorded at the prepared biosensor in 0.1 M sodium phosphate buffer, pH 6.5 at scan rate of 100 mV/s before (a) and after (b) addition of 20 mM of glucose.

The electrochemical response of the modified electrodes toward glucose was evaluated by cyclic voltammetry. As shown in Figure 2, in the absence of the monosaccharide, the electrode exhibited a typical voltammetric profile of a Fc-containing surface, with a pair of well-defined redox peaks characterized by E_1/2_ = 250 mV, ΔE_p_ = 112 mV, and i_pa_/i_pc_ = 3.46. These features suggest a quasi-reversible redox process associated with the pendant Fc moieties covalently attached to the polymer backbone.

In the presence of glucose, this voltammetric behavior changed markedly, with a pronounced increase in the anodic current in the potential region corresponding to Fc oxidation and a concomitant decrease in the cathodic peak intensity. This behavior can be rationalized according to the following electrocatalytic mediation mechanism:

Glucose + GOx(FAD) + H_2_O → Gluconic acid + GOx(FADH_2_)

GOx(FADH_2_) + 2Fc^+^ → GOx(FAD) + 2Fc + 2H^+^

2Fc → 2Fc^+^ + 2e^−^ (electrode reaction)

As depicted in these reactions, the reduced form of GOx, generated after enzymatic oxidation of glucose, is reoxidized by the electrochemically generated ferrocenium species. This process regenerates Fc at the electrode surface, enabling its repeated electrochemical oxidation and producing an amplified anodic response.

**Figure 3:**
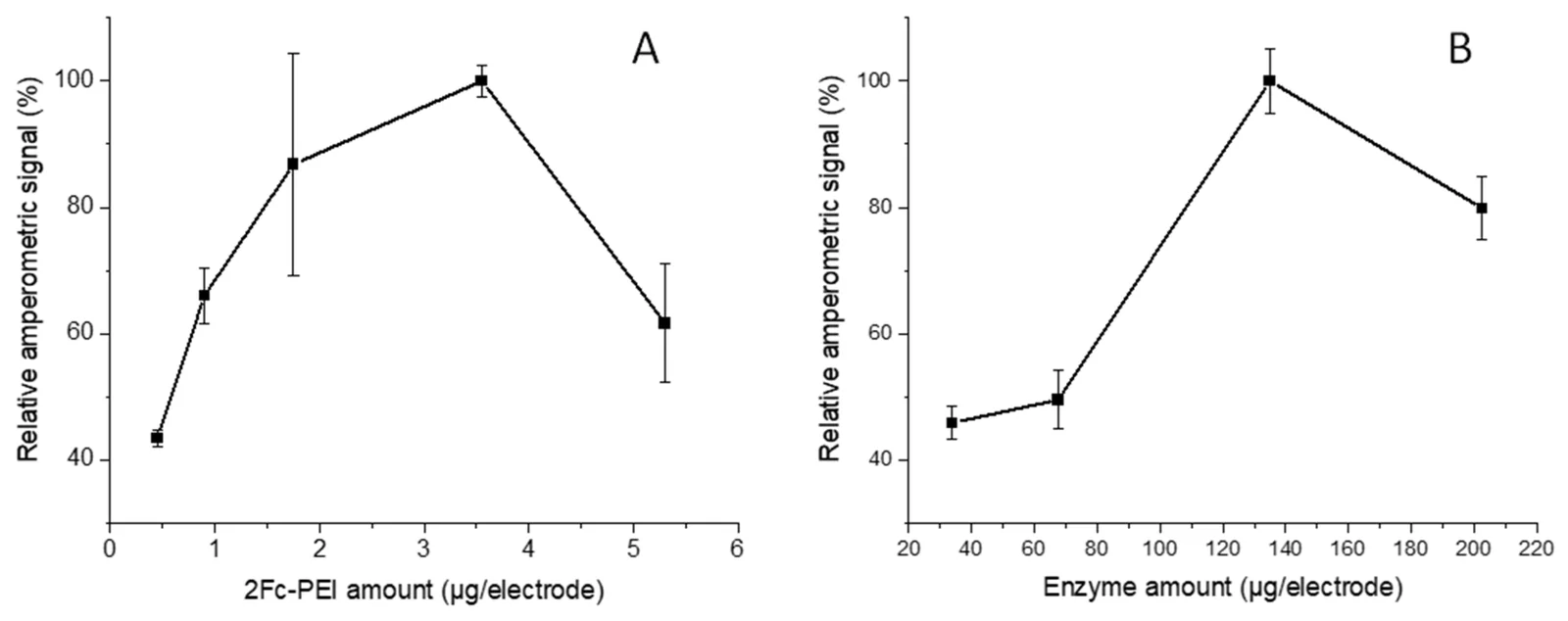
Influence of the 2Fc-PEI amount (**A**) and GOx amount loading (**B**) on the amperometric signal in response to 20 mM of glucose in 0.1 M sodium phosphate buffer, pH 6.5. Working potential = 400 mV.

The experimental conditions for biosensor fabrication were optimized by assessing the influence of the different assembly parameters on the amperometric response of the enzyme-functionalized electrodes toward 20 mM glucose. All optimization experiments were performed in triplicate. The relative amperometric signal was evaluated by comparing each individual amperometric signal with the highest signal obtained under the specified experimental conditions, which was assigned a value of 100%. The amount of Fc-branched polymer incorporated into the drop-cast film was first optimized. As shown in Figure 3A, the analytical response increased with polymer loading, reaching a maximum at 3.5 µg per electrode. Above this value, the current decreased, most likely because an excessive polymer content increases the diffusion barrier within the film, thereby restricting glucose access to the enzyme active sites. Therefore, 3.5 µg of polymer per electrode was selected for subsequent experiments.

The loading of GOx was also optimized. As shown in Figure 3B, the amperometric response increased with the enzyme loading up to 135 µg per electrode, which provided the highest current response, and was therefore selected for subsequent experiments. Lower GOx loadings likely resulted in reduced catalytic activity within the film, whereas higher enzyme amounts did not further improve the response. This effect may be ascribed to the formation of an excessively compact enzyme layer, which could decrease the apparent specific activity of the immobilized GOx due to steric hindrance between neighboring enzyme molecules and restricted substrate accessibility to the active sites.

The operational measurement conditions for this biosensor were also optimized by determining the effect of working potential and pH on the amperometric response of this analytical device. As can be observed in Figures S4 and S5 in Supporting Information, best results were achieved by measuring at 400 mV in 100 mM sodium phosphate buffer, pH 6.50. The optimum pH observed experimentally is consistent with the value reported by the commercial supplier for GOx from Aspergillus niger, for which an optimum pH of 6.5 is indicated. This behavior can be attributed to the dependence of GOx activity on the protonation state of amino acid residues involved in substrate recognition and catalysis, which affects the enzymatic conversion of glucose under the different pH conditions.

**Figure 4:**
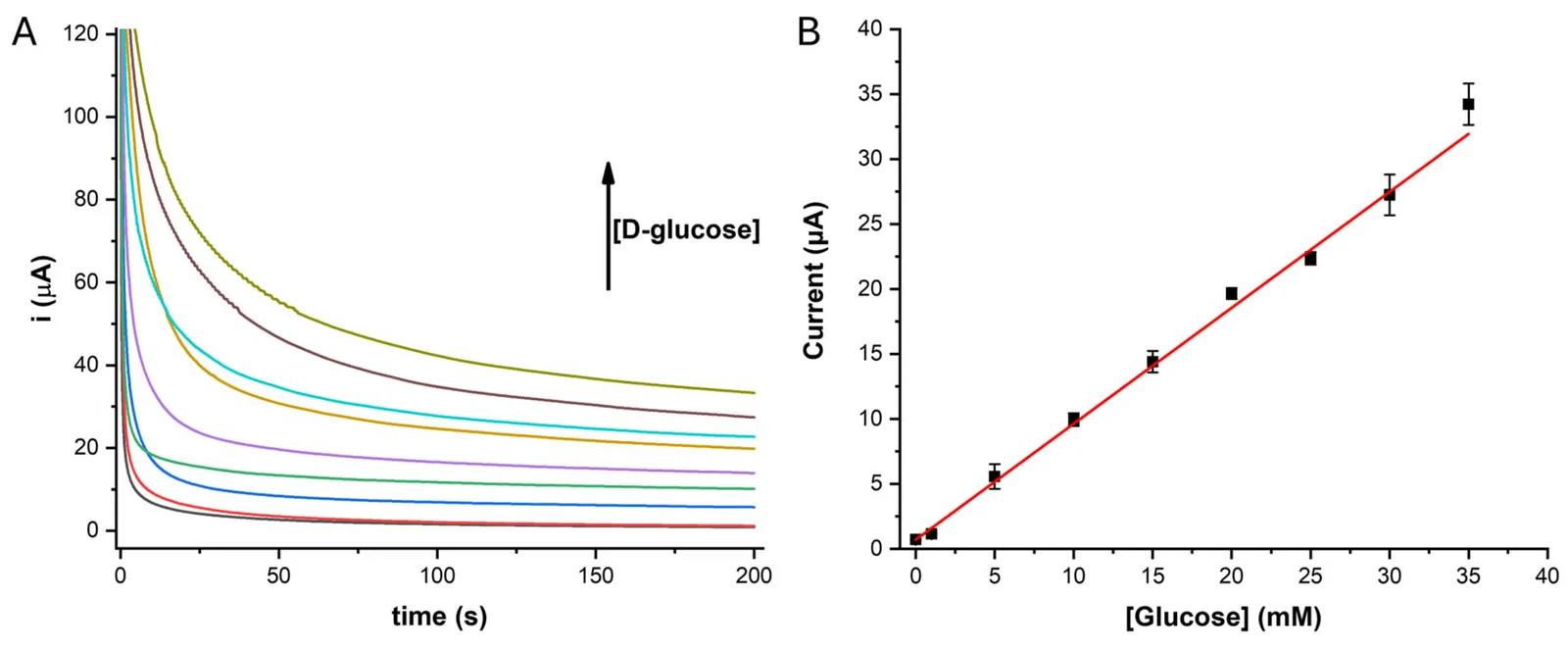
Amperograms (**A**) and calibration plot (**B**) obtained with the prepared biosensors toward glucose recorded at 400 mV and 200 s, in 0.1 M sodium phosphate buffer, pH 6.5 containing glucose at different concentrations: 0 mM, 1 mM, 5 mM, 10 mM, 15 mM, 20 mM, 25 mM, 30 mM and 35 mM.

Under these optimized conditions, the analytical performance of the enzyme biosensor toward glucose in buffered solutions was then evaluated. The analytical device exhibited an adequate response, reaching 95% of the steady-state current within 50 s (Figure 4A). As is illustrated in Figure 4B, the biosensor showed a linear relationship between amperometric signal, obtained from the anodic current associated with the oxidation at 400 mV of the Fc moieties on the branched polymer, and the concentration of glucose between 0 mM and 35 mM, according to the following equation (r^2^ = 0.995, *n* = 10):

i (µA) = (0.92 ± 0.02)·[glucose, mM] + (0.6 ± 0.5)

where the uncertainty denotes the standard deviation of slope and ordinate intercept.

The sensitivity was estimated from the slope of the linear calibration plot as 0.92 µA/mM, and the limit of detection as 33 µM according to the IUPAC rules [33].

The linear range of the proposed biosensor was compared with reported glucose concentrations in commercial wines. Reported concentrations ranged from 1.89 to 95.19 mM in commercial white wines [34], while concentrations of 2.05 and 14.60 mM have been reported for commercial red and white wines, respectively [35]. These data indicate that the proposed biosensor is suitable for many wines, although samples with higher glucose concentrations may require appropriate dilution prior to analysis.

The reproducibility of the biosensor preparation was evaluated by comparing the amperometric response of 10 independently prepared electrodes fabricated following the same procedure on different days toward a 0.023 M glucose solution. A relative standard deviation (RSD) of 2.7% was obtained, indicating excellent reproducibility of the biosensor preparation.

**Figure 5:**
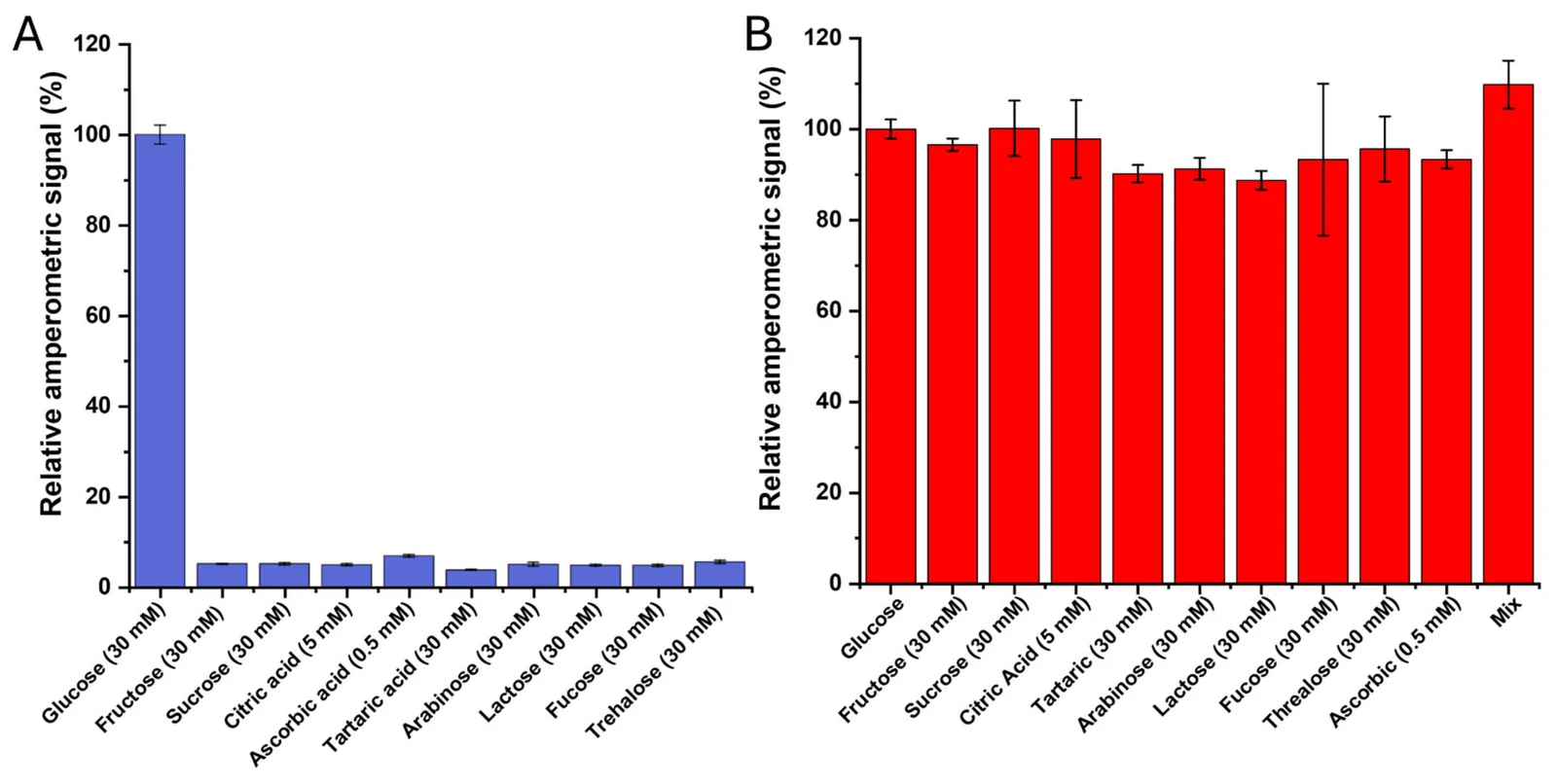
Relative amperometric response of (**A**) glucose and individual interfering species and (**B**) a mixture of glucose and the indicated interfering species at the biosensor.

Selectivity studies for this biosensor were performed by testing its amperometric response toward nine different compounds commonly found in food samples. The potential interfering species were tested at the following concentrations: fructose, sucrose, arabinose, lactose, fucose, and trehalose at 30 mM; citric acid and tartaric acid at 5 mM; and ascorbic acid at 0.5 mM. In the interference study, relatively high concentrations of the tested compounds were intentionally selected to provide a stringent evaluation of the selectivity of the biosensor. For the interference study in the presence of glucose (Glu), 30 mM glucose was used together with each interfering species at the concentrations indicated above. The relative amperometric signal was calculated by assigning 100% to the amperometric signal obtained for 30 mM glucose, while the signals obtained for the interfering species were expressed relative to this value. As can be observed in Figure 5A, relative amperometric signal measured towards these compounds represented less than 10% of the signal corresponding to glucose. Accordingly, we can conclude that the enzyme biosensor exhibited excellent selectivity. In a further set of experiments, the potential interference of these compounds was assessed by measuring the response of the reagentless biosensor toward glucose solutions containing the tested species. As shown in Figure 5B, the presence of these compounds did not produce any significant variation in the glucose signal, indicating that they exert negligible interference on the electroanalytical determination of glucose.

**Figure 6:**
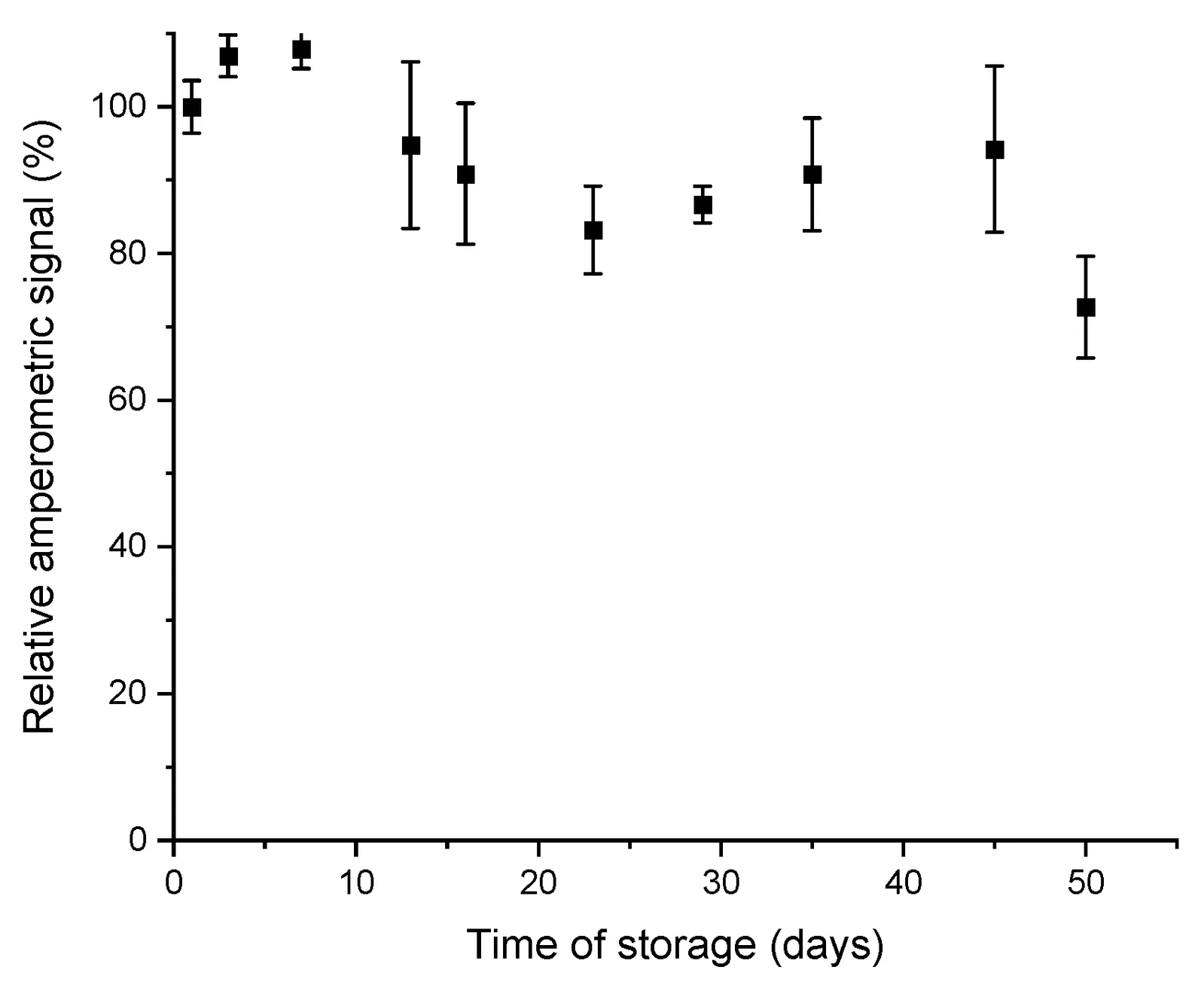
Effect of time of storage at 4 °C on the relative amperometric response of the biosensor toward 20 mM of glucose.

Figure 6 shows the effect of storage duration at 4 °C under dry conditions on the analytical response of the enzyme biosensor toward glucose. The device exhibited high long-term stability, retaining more than 80% of its initial response after 45 days. This behavior suggests that the immobilized enzyme preserved a significant fraction of its structural and functional integrity, likely due to the stabilizing effect of bovine serum albumin and Nafion within the film. In addition, the cross-linked matrix appears to provide sufficient mechanical stability to minimize film detachment from the electrode surface and prevent leaching of the bioactive components. In general, this enzyme biosensor showed similar or better analytical performance than other devices previously reported, in terms of linear range of response, sensitivity and stability, as can be appreciated from Table 1. The limit of detection obtained in the present work (33 µM) is also in the range of values reported for Fc-based glucose biosensors, although lower LOD values have been reported for some devices [36,37,38,39] (Table 1). The analytical performance of the proposed biosensor may be related to the incorporation of redox-active Fc moieties into the PEI-based polymeric network, which can facilitate electron transfer between the enzymatic reaction and the electrode. Moreover, the polymeric matrix provides a favorable environment for GOx immobilization, while the cross-linked film helps retain the enzyme at the electrode surface. These features may promote efficient electrochemical transduction of the enzymatic reaction. Although some previously reported systems exhibit lower LOD values, the proposed biosensor offers a broad linear range (0–35 mM), good storage stability (>45 days), a straightforward fabrication strategy based on a one-pot Ugi reaction and drop-casting, and a reagentless format using disposable SPCE. These characteristics are relevant for practical glucose determination in food samples.

The applicability of the electroanalytical device was finally validated through the determination of total glucose content in commercial red and white wine samples. As shown in Table 2, the glucose concentrations obtained with the amperometric biosensor were in good agreement with those determined using a commercial enzymatic kit based on colorimetric detection. No statistically significant differences were observed between the results obtained by the two methods (Welch’s *t*-test, *p* > 0.05). These results demonstrate the reliability of the proposed device for glucose determination in complex real food samples.

## 4. Conclusions

In this work, a novel Fc-branched polyethyleneimine (2Fc-PEI) derivative was successfully synthesized through a Ugi multicomponent reaction and employed as a redox-active matrix for the development of a reagentless amperometric glucose biosensor. The incorporation of Fc moieties into the PEI backbone was supported by spectroscopic, elemental, and thermal characterization. The resulting GOx/2Fc-PEI-based biosensor exhibited a linear response to glucose from 0 to 35 mM, with a sensitivity of 0.92 µA/mM and a limit of detection of 33 µM. The biosensor reached 95% of the steady-state response within approximately 50 s and showed good selectivity toward potential interfering compounds. In addition, the device retained more than 80% of its initial response after 45 days of storage at 4 °C, demonstrating good storage stability. Finally, the biosensor was applied to glucose determination in commercial red and white wine samples, yielding results in good agreement with those obtained using a commercial enzymatic colorimetric assay. These results indicate that the proposed biosensor may be suitable for glucose determination in wine samples. Compared with previously reported Fc-based glucose biosensors, the proposed device combines a broad linear range, low detection limit, good storage stability, and a reagentless format using a disposable SPCE. These features, together with its successful application to wine samples, highlight the potential of the proposed platform for practical glucose determination in food analysis and its possible applicability in routine and decentralized wine-quality control.

## Figures and Tables

**Table 1:** Comparison of the analytical performance of relevant glucose enzyme biosensors using Fc derivatives.

Sensor	Electrochemical Technique	Linear Range (mM)	LOD (µM)	Stability (Days)	Sensitivity (µA/mM)	Reference
Fc–PLL/GOx	CA	0–10	23	-	6.8	[17]
GOx/Fc-Chit/CNTs/SPE	CV	2 × 10^−2^–8 × 10^−1^	20	21	8.5	[36]
MWCNT–Fc/GOx–Nafion/GCE	CA	2.8 × 10^−1^–15	9	35	1.52	[37]
GOx/Fc-NH_2_-Hf-BTB-MOL/C-CNTs	CA	5 × 10^−3^–4 × 10^−1^ 3–9	3.9	-	6.92	[38]
PG/Nafion/Fc/GOx-GCE	EIS	1–15	21	50	10.6 kΩ/mM	[39]
BSA/Fc/SWCNT–COOH/GO–FAD	CA	5 × 10^−2^–2.3	50	10	-	[40]
GDH/LPEI-Fc/GO/SPE	CA	1–40	280	-	0.07	[41]
SPE/CTT–Fc/GOx/Chit	CA	0–10	190	30	0.68	[42]
SPCE/PEI–2Fc–GOx–Nafion–BSA (this work)	CA	0–35	33	45	0.92	-

**CA**: chronoamperometry; **CV**: cyclic voltammetry; **EIS**: electrochemical impedance spectroscopy; **GOx**: glucose oxidase; **Fc**: ferrocene; **Chit**: chitosan; **CNTs**: carbon nanotubes; **SPE**: screen-printed electrode; **PLL**: poly-L-lysine; **GDH**: glucose dehydrogenase; **LPEI**: linear poly(ethylenimine); **GO**: graphene oxide; **MWCNT**: multi walled carbon nanotubes; **GCE**: glassy carbon electrode **CTT**: carbonised Typha tassel; **Hf**: hafnium; **BTB**: 1,3,5-tribenzoic acid; **MOL**: metal–organic layers; **C-CNTs**: carboxylated carbon nanotubes; **PG**: porous graphene.

**Table 2:** Determination of glucose in commercial wine samples. Results are expressed as mean ± standard deviation (*n* = 3).

Sample	Glucose [g/L]	
	Colorimetric	Electrochemical
Chulato de Abeica red wine	0.29 ± 0.03	0.30 ± 0.02
Don Simón Chardonnay-Airén dry white wine	1.546 ± 0.003	1.5 ± 0.2

## Data Availability

Data are contained within the article and Supplementary Materials.

## Supplementary Materials

The following supporting information can be downloaded at https://www.mdpi.com/article/10.3390/bios16100554/s1, Figure S1: Solid-state ^13^C NMR spectra of 2Fc-PEI recorded at a 12 kHz magic-angle spinning rate; Figure S2: FT-IR spectra of PEI (a), Fc-carboxylic acid (b), Fc-carboxaldehyde (c) and 2Fc-PEI (d); Figure S3: TG Analysis of PEI (a) and 2Fc-PEI (b); Figure S4: Influence of pH on the amperometric signal in response to 20 mM of glucose in 0.1 M sodium phosphate buffer. Working potential = 400 mV. Figure S5. Influence of working potential on the amperometric signal in response to 20 mM of glucose in 0.1 M sodium phosphate buffer, pH 6.5.
