# Supplementary material for: Ferrocene-Branched Polymers via a Ugi Multicomponent Reaction for an Integrated and Reagentless Amperometric Glucose Biosensor
Source: Biosensors (Basel). 2026 Oct 2;16(10):554. doi: 10.3390/bios16100554 (PMC13702432; doi:10.3390/bios16100554)
Supplement: Supplementary file 1 [file biosensors-16-00554-s001.zip › biosensors-4487616-supplementary.pdf]

Supporting Information for

Ferrocene-Branched Polymers via a Ugi Multicomponent Reaction for an Integrated and Reagentless Amperometric Glucose Biosensor

Jorge Urraca, Isabel Zurita, Marija Krznaric, Tatiana Martínez, Alfredo Sánchez, Diana Vilela, Paloma Martínez-Ruiz, Irene Ojeda * and Reynaldo Villalonga *

Nanosensors and Nanomachines Group, Department of Analytical Chemistry, Faculty of Chemistry, Complutense University of Madrid, 28040 Madrid, Spain; jorgeurr@ucm.es (J.U.); isabel.zurita@estudiante.uam.es (I.A.Z.); mkrznaric1.chem@pmf.hr (M.K.); tati3520@gmail.com (T.M.); alfredos@ucm.es (A.S.); divilela@ucm.es (D.V.); palmarti@ucm.es (P.M.-R.)

*** Correspondence: ireojeda@ucm.es (I.O.); rvillalonga@quim.ucm.es (R.V.)**

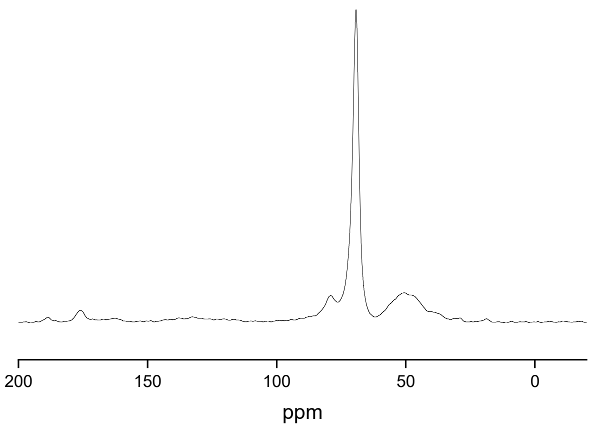

Figure S1. Solid-state ^{13}C NMR spectra of 2Fc-PEI recorded at a 12 kHz magic-angle spinning rate

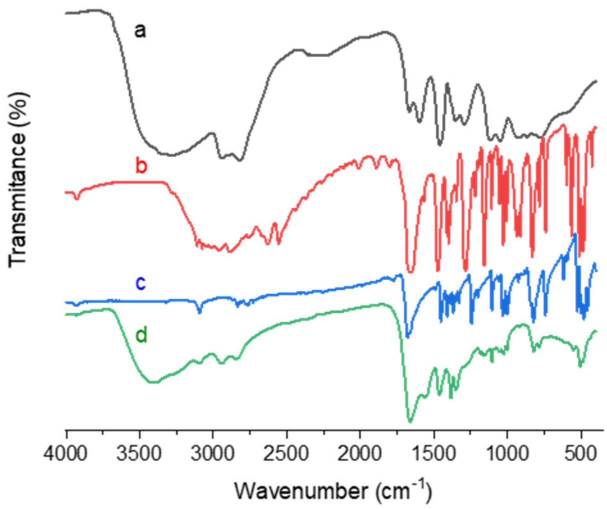

Figure S2. FT-IR spectra of PEI (a), ferrocenecarboxylic acid (b), ferrocenecarboxaldehyde (c) and 2Fc-PEI (d)

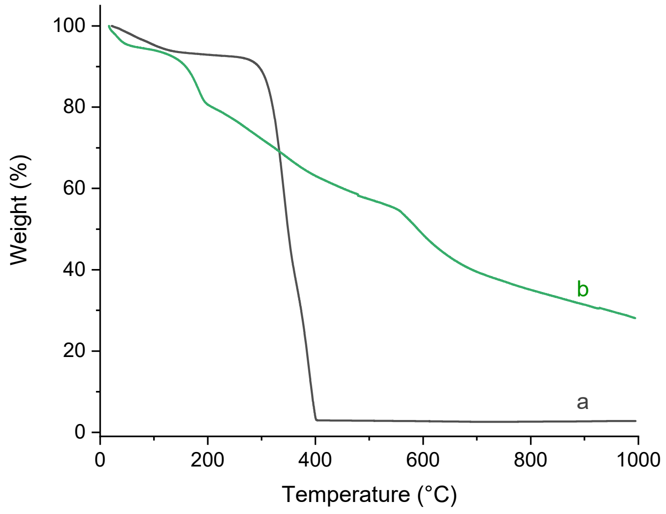

Figure S3. Thermogravimetric analysis of PEI (a) and 2Fc-PEI (b)

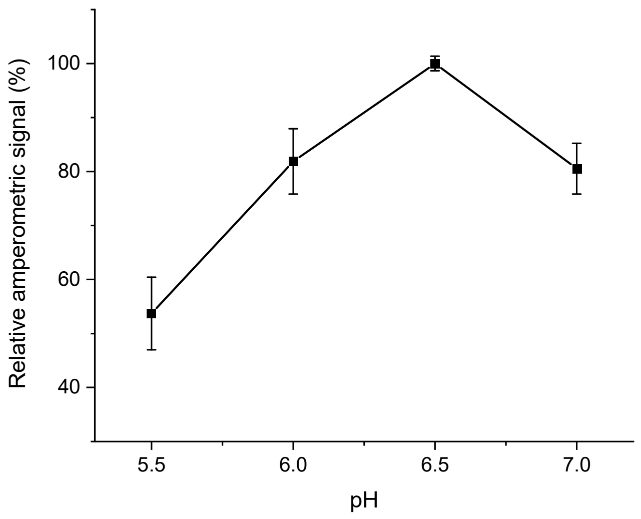

Figure S4. Influence of pH on the amperometric signal in response to 20 mM of glucose in 0.1 M sodium phosphate buffer. Working potential = 400 mV.

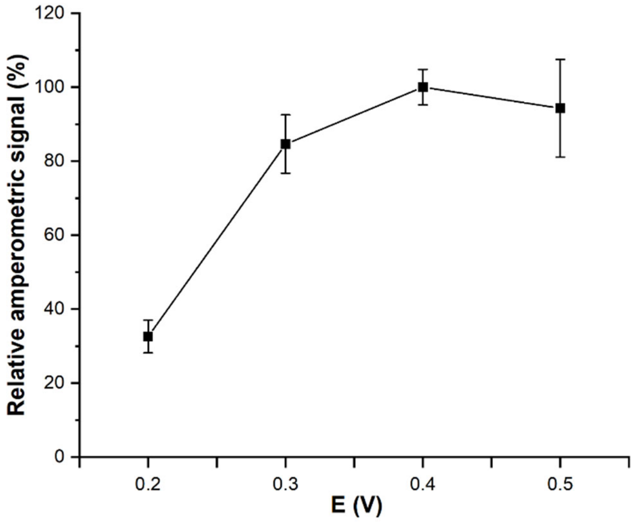

Figure S5. Influence of working potential on the amperometric in response to 20 mM glucose in 0.1 M sodium phosphate buffer at pH 6.5.
